# Effects of waterlogging on pollutant availability in iron mining tailings and the tolerance response of *Typha domingensis* Pers

**DOI:** 10.1007/s11356-026-38164-2

**Published:** 2026-08-25

**Authors:** Marcelo Ramos de Anchieta, Kauê Shindi Dias Nakamura, Amanda Coletti Santolino, Carlos Henrique Goulart dos Reis, Vitor Hugo Silva Ribeiro, Vinícius Politi Duarte, Fabricio José Pereira

**Affiliations:** 1 Instituto de Ciências da Natureza, Universidade Federal de Alfenas, Alfenas, Minas Gerais State Brazil; 2 Instituto de Ciências Naturais, Universidade Federal de Lavras, Lavras, Minas Gerais State Brazil

**Keywords:** Root anatomy, Phytoremediation, Mining wastes, Cattail, Metal pollutants

## Abstract

Soil flooding can increase nutrient and pollutant availability to plants; however, the effects in iron mining tailings are still unclear. This work hypothesizes that waterlogging increases the availability of elements present in iron mining tailings, but tolerant aquatic plants can survive under these conditions, showing potential for phytoremediation. This work investigated how flooding affects the availability of essential elements and metal pollutants in iron mining tailings and how this affects the growth, root anatomy, and pollutant content in the aquatic plant *Typha domingensis* (cattail). Cattail clones were grown in pots containing iron mining tailings under two irrigation conditions: field capacity and waterlogged. The experimental design was completely randomized with 18 replicates and two treatments (*n* = 36), and the experiment was conducted for 180 days. The pH, electrical conductivity, and total dissolved solids were measured from the iron mining tailings and the plant contents of Al, Cd, Cr, Pb, Fe, and Ca, as well as their growth and root anatomy were evaluated. Waterlogging increased the pH (from 6 to 7), total dissolved solids (from 61.9 to 140.8 ppm), and the electrical conductivity (from 113.2 to 282.1 µs cm^−1^) of iron mining tailings, suggesting higher ion availability in the water. Waterlogging of iron mining tailings also increased the elemental uptake, ranging from 50 to 178% depending on the element considered. No growth limitation was observed since waterlogging also increased the root dry and fresh masses, the root:shoot ratio, the root biomass allocation, and plant dry mass from cattail. Waterlogging of iron mining tailings also increased the root cortex area, the exodermis thickness, and the vascular cylinder area. Results suggest that waterlogging of iron mining tailings increases the availability and uptake of elements by *T. domingensis*, which, nevertheless, suffers no significant growth limitations and shows relevant potential for the remediation of iron mining tailings when the pollutant is present in natural wetlands and when the species is used in remediation systems.

## Introduction

Several regions in the world have been suffering from the mining tailings dam failures, and such events were reported in Europe (Rico et al. 2008), Asia (Lin et al. 2022), and the Americas (Carmo et al. 2017). Regarding iron mining tailings, one of the biggest dam failures occurred at Mariana municipality, Minas Gerais state, Brazil, in November 2015, releasing 50 million m^3^ of tailings in the Doce River Basin, causing environmental damage (Lacaz et al. 2017; Carmo et al. 2017). Iron mining tailings released in this failure showed several pollutants such as aluminum (Al), cadmium (Cd), lead (Pb), chromium (Cr), and high levels of iron (Fe) (Gomes et al. 2025). The impact is huge, and despite some plant species being reported as tolerant to iron mining tailings (Gomes et al. 2025), all plants indicated for remediation of this pollutant are trees and herbaceous plants adequate for regions previously containing drained areas. Several areas along the Doce River Basin are subjected to waterlogging, and a large part of the tailings arrived at the river (Lacaz et al. 2017; Carmo et al. 2017). Waterlogged environments promote several secondary stresses to plants, such as the reduction of O_2_ availability to roots (Manghwar et al. 2024), which require aerenchyma development to overcome this limitation (Duarte et al. 2021). This tissue is often absent in plants from drained conditions, limiting their use in waterlogged systems. However, no information is available about either the physiological tolerance or phytoremediation potential of aquatic plants to iron mining tailings or how waterlogging could affect the availability of pollutants.

Waterlogging can be a secondary stress factor, and plants adapted to drained regions could not stand an additional stressor. It was already shown that secondary factors, such as the pH value, can increase the toxicity of iron mining tailings for sensitive species (Gomes et al. 2025), and compaction of iron mining tailings reduced seed germination and some growth parameters in seedlings of tolerant trees (da Silva et al. 2025). Waterlogging was never explored as a secondary factor affecting plants growing in iron mining tailings. Nonetheless, climate change is increasing the frequency of flooding and waterlogging events (Cui et al. 2022). Waterlogging can be a strong stressor for plants because of the reduced O_2_ availability to the root system (Manghwar et al. 2024) and because water acts as a solvent for ionic pollutants present in the iron mining tailings (Ponting et al. 2021; Yu et al. 2025), such as Al^3+^_,_ Cd^2+^, Cr^3+^, Fe^2+^, and Fe^3+^. The lower O_2_ availability requires studies with aquatic plants such as *Typha domingensis* Pers., which develops aerenchyma (Duarte et al. 2021), that tolerate such conditions, and the capacity of water to solubilize ionic pollutants can increase their availability to plants, intensifying the iron mining tailings toxicity.

*Typha domingensis* Pers. is an aquatic plant found worldwide and was already described as tolerant to Cd (de Oliveira et al. 2022), Cr (Mufarrege et al. 2015), and Pb (Bonanno 2013). This species was also reported as tolerant to gold mining tailings (Compaore et al. 2020). Thus, this species potentially is tolerant to iron mining tailings, despite this aspect having never been investigated yet. Being an aquatic plant, *T. domingensis* shows adaptations to deal with low O_2_ availability to the root system, such as aerenchyma development and possibly O_2_ production in roots from the scavenging of H_2_O_2_ by the action of the Catalase enzyme (Duarte et al. 2021). These adaptations can alleviate the secondary stresses imposed by waterlogging, permitting plant energy to be used in dealing with iron mining tailings pollutants more efficiently, since they could be more available to be absorbed.

Roots can be one of the most sensitive organs to soil pollutants because of their direct contact with the substrate and their function to uptake water and nutrients. Roots show apoplastic barriers in the endodermis and exodermis that deposit mainly suberin in their cell walls, hampering the apoplastic flow, and avoiding pollutants reaching the xylem and being transported to shoots, and protecting the photosynthetic system (Doblas et al. 2017; de Oliveira et al. 2022; Liu and Kreszies 2023). Roots can then store pollutants in their cortical cells (Xu et al. 2017), thereby avoiding their transport to shoots and protecting the photosynthetic system. *T. domingensis* roots showed the capacity to increase their apoplastic barriers under Cd contamination, being an important tolerance mechanism for the species (de Oliveira et al. 2022); nonetheless, root anatomical traits of *T. domingensis* were never investigated when it is growing in mining tailings.

This work hypothesizes that waterlogging increases the availability of essential elements and pollutants present in iron mining tailings, but that tolerant aquatic plants can survive under these conditions, showing potential for phytoremediation. Thus, this work aimed to investigate the effects of waterlogging of iron mining tailings on the essential elements and uptake, growth, and root anatomical traits of the aquatic plant *T. domingensis*.

## Material and methods

### Plant material

*T. domingensis* plants were collected from a natural population located in Alfenas, Minas Gerais state, Brazil (21º24′06.2″ S and 45º59′07.5″ W). These plants were washed in tap water and then cultivated in a greenhouse located at the Universidade Federal de Alfenas. Plants were grown in pots containing 20 L of nutrient solution (Hoagland and Arnon 1950). These plants were grown for 60 days to obtain clones that were acclimated to the greenhouse conditions and that were used in experiments. Water lost by evapotranspiration was replaced daily, and the nutrient solution was replaced fortnightly.

### Experimental design

Clones acclimated to the greenhouse were selected by similar size (50 cm in height and four leaves, containing rhizomes and roots). Clones were transferred to pots containing 2 L of iron mining tailings previously prepared to receive two irrigation conditions: field capacity and waterlogged. Field capacity was considered when iron mining tailings were saturated by water and left to be drained to their maximum water-holding capacity (still containing some air in tailings’ pores), which was achieved with the application of 800 mL of water to the 2 L of tailings. Iron mining tailings were sieved through a 2.5 mm mesh before being added to the pots. Waterlogging was promoted by applying water to the iron mining tailings until a water layer of 3 cm depth was permanently formed above the tailings surface. Water availability was maintained by replacing the water lost by evapotranspiration daily, which requires about 100 mL per day for the field capacity treatment, and by adding water until reaching a mark indicating 3 cm of depth for the waterlogged treatment. Each treatment (water conditions) received 18 replicates, with a total of 36 parcels (*n* = 36), each parcel comprised of one pot, which received one *T. domingensis* clone. Experiments were conducted in a greenhouse at 25 ± 2 ºC and 187 W m^−2^ for 180 days.

### Iron mining tailings sampling and analysis

Iron mining tailings were collected 4.0 km from the Fundão dam in Mariana, state of Minas Gerais, Brazil (20° 11′ 58″ S and 43° 29′ 28″ W), at a depth of 1 m; further, they were transported to the Universidade Federal de Alfenas and stored in plastic bags and in a room protected from humidity and direct radiation until the start of the experiments.

The concentrations of macronutrients phosphorus (P), magnesium (Mg), potassium (K), and calcium (Ca), and micronutrients manganese (Mn), iron (Fe), zinc (Zn), copper (Cu), and sodium (Na) as well as the potentially toxic elements (PTEs) aluminum (Al), cadmium (Cd), chromium (Cr), and lead (Pb) were determined in the tailings before starting the experiments and results are shown in Table 1.

**Table 1:** Chemical analysis of the iron mining tailings before the installation of the experiments, containing macro and micronutrients, potentially toxic elements, and the pH value

Macronutrients		mg Kg^−1^
P	232.1	
Mg	115.7	
K	128.3	
Ca	5736.5	
Micronutrients	mg Kg^−1^	
Mn	432.7	
Fe	29936.15	
Zn	8.8	
Cu	6.1	
Na	72.0	
Potentially toxic elements	mg Kg^−1^	
Al	2385.6	
Cr	11.7	
Cd	2.4	
Pb	6.8	
Other traits		
pH	6.0	

The tailings analysis was performed using the protocol from AOAC (2005). Mining tailings were oven-dried at 60 °C until reaching a constant mass and submitted to different extraction methods for elemental quantification. For this, 500 mg of the tailing separated, and 50 mL of KCl was applied for Ca, Mg, and Al solubilization, while the K and PO_4_^−^ contents were extracted with 50 mL of 0.025 mol L^−1^ H_2_SO_4_ + 0.05 mol L^−1^ HCl. Micronutrients were measured using a chelating solution, and PTEs were extracted after nitroperchloric digestion. The levels of macro- and micronutrients and potentially toxic elements were quantified using an atomic absorption spectrophotometer, AAnalyst 800 (PerkinElmer, Waltham, MA, USA) (Abbruzzini et al. 2014).

The pH of the iron mining tailings was measured using a portable pH meter model HPH-125H (Homis, São Paulo, Brazil). The total dissolved solids (TDS) and electrical conductivity were measured using a portable conductimeter model AK83 (Akso, Rio Grande do Sul, Brazil). To measure interstitial water parameters in the field capacity treatment, a small cavity (2 cm diameter and 5 cm depth) was created to allow for the accumulation of pore water, into which the electrodes were inserted. For the waterlogged treatment, measurements were performed directly in the water, introducing the electrode close to the tailings surface (approximately 3 cm depth). The pH, TDS, and electrical conductivity were measured monthly in the morning, and the water temperature was 17 ± 2 ºC. The data were averaged before analysis.

### Growth parameters

Plants were collected at the end of the experiment (180 days), being removed from the iron mining tailings and washed in tap water, and then separated into roots and shoots and weighed on an analytical scale AY 220 (Shimadzu, Tokyo, Japan) for the fresh mass quantification. Two roots per plant were photographed, and their length was measured using the ImageJ software version 1.45 s (Schneider et al. 2012). The root diameter was measured at the root base, middle, and apex using a digital pachymeter (Mitutoyo, Jundiaí, Brazil).

Plants were oven-dried at 60ºC for seven days until constant mass, and then the total dry mass, the shoot, and root dry masses were measured in an analytical scale AY 220 (Shimadzu, Tokyo, Japan). The root:shoot ratio was calculated by dividing the root dry mass by the shoot dry mass. The root biomass allocation was calculated as follows: AL% = (root dry mass/total dry mass)*100, where AL% is the proportional allocation of biomass to roots. The root water content was calculated as follows: RW% = [(root fresh mass – root dry mass)/root fresh mass]*100, where RW% is the root water content.

### Quantification of essential elements and pollutants in plants

At the end of the experiment (180 days), the plants collected were oven-dried at 60ºC for seven days and kept in plastic bags until analysis. The quantification method was performed according to AOAC (2005). For this analysis, 500 mg of plant material was oven-dried at 60 °C for 72 h and then transferred to tubes containing 6 mL of HNO_3_ and kept at 25ºC overnight. Tubes were then placed in a digestion block and heated to 140 °C. Samples were kept at this temperature until the volume of the solution was reduced to 1 mL. Samples were cooled, and then 2 mL of HClO_4_ was added, and the solution was heated to 190 °C for 2 h (Abbruzzini et al. 2014). Samples were then cooled, and the quantification of Al, Ca, Cd, Cr, Fe, and Pb was performed in an atomic absorption spectrometer AAnalyst 800 (PerkinElmer, Waltham, MA, USA).

### Anatomical analysis

Two roots per plant were collected for anatomical analysis and immediately fixed in 70% ethanol (V V^−1^). Transversal sections were hand-made using steel blades at a distance of 2 cm from the root apex. Sections were clarified in 50% sodium hypochlorite (V V^−1^) for 10 min and washed twice in distilled water for 10 min. Sections were then stained with a safrablau solution [1% safranin (m V^−1^) and 0.1% Astra blue (m V^−1^) at the proportion of 3:7] (Johansen 1940). Sections were placed in slides containing 50% glycerol (V V^−1^) and covered with coverslips. Slides were observed and images captured in an Eclipse *Si* microscope (Nikon, Tokyo, Japan), and images were analyzed with the ImageJ software version 1.45 s (Schneider et al. 2012). For each root, one slide was made, and for each slide, three sections were evaluated (totalizing 54 sections per treatment and 108 sections for the experiment); data were averaged for each replicate. The anatomical traits evaluated were: epidermis thickness, exodermis thickness, cortex thickness, endodermis thickness, xylem vessel diameter from proto and metaxylem, aerenchyma chamber area, vascular cylinder area, and xylem vessel element area.

### Statistical analysis

Data was submitted to the Shapiro–Wilk test for normal distribution, and data from all variables showed normal distribution. Data were then submitted to ANOVA and to the Scott-Knott test at *P* = 0.05. Statistical analyses were performed with the Sisvar software version 5.6 (Ferreira 2011).

## Results

**Fig. 1:**
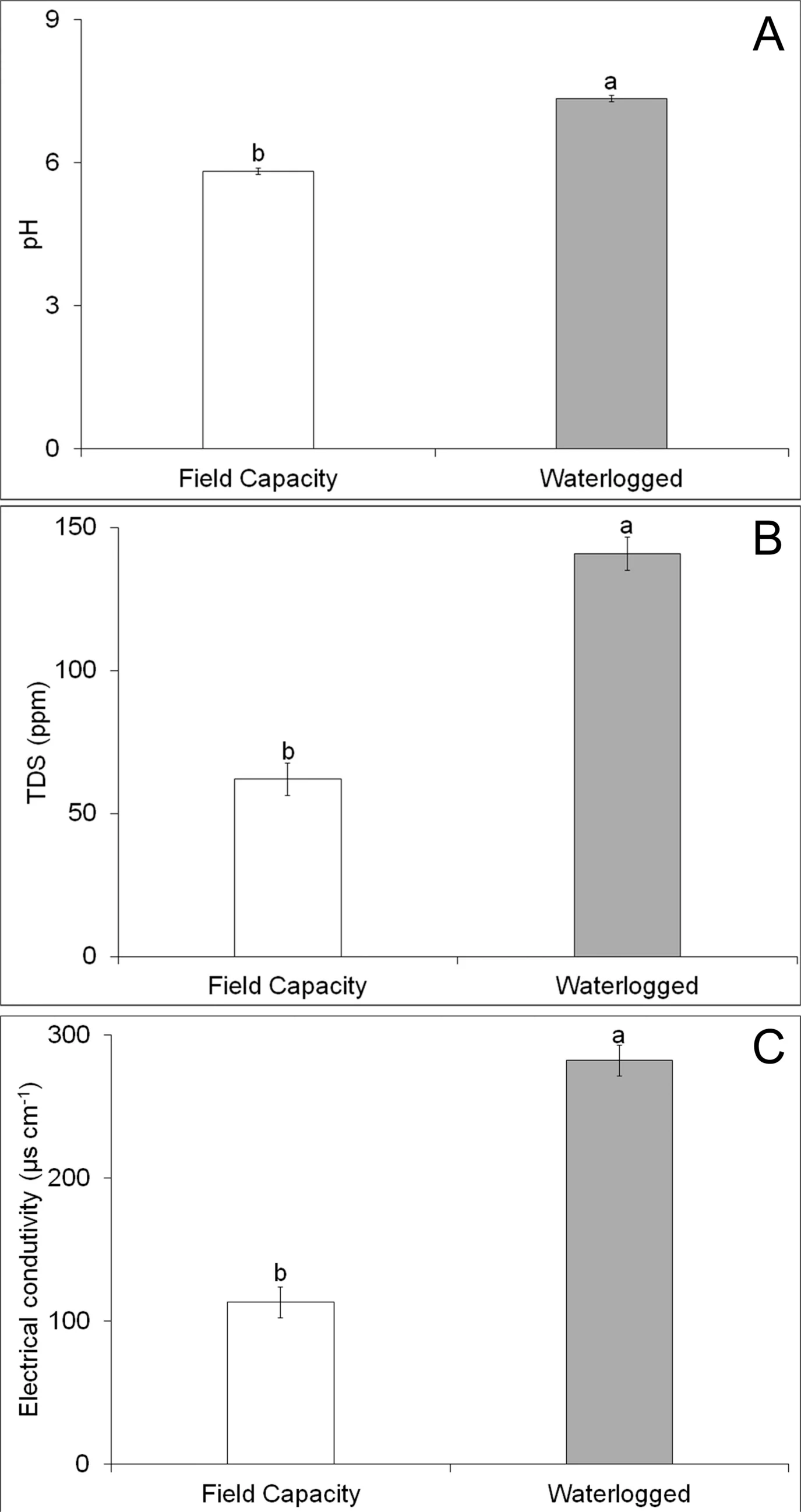
Physicochemical parameters from iron mining tailings under different irrigation conditions. pH (**A**), total dissolved solids (TDS) (**B**), and electrical conductivity (**C**). Different letters in columns indicate significant differences according to the Scott-Knott test, *P* < *0.05* (*n* = 18). Bars = standard error

Waterlogging increased the pH by 26.9% (Fig. 1A), total dissolved solids by 120.3% (TDS) (Fig. 1B), and electrical conductivity by 149.6% (Fig. 1C) of iron mining tailings.

**Fig. 2:**
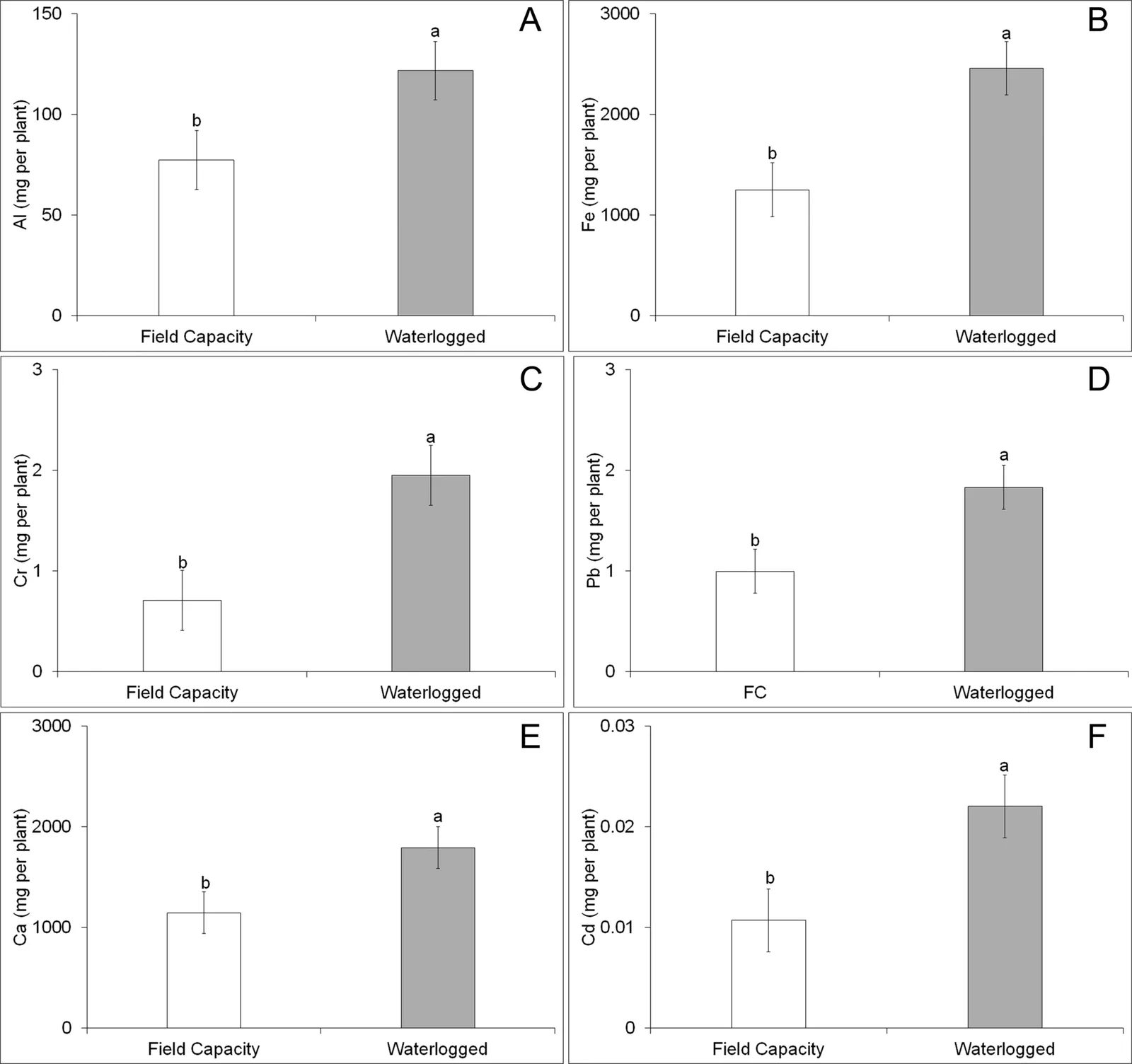
Concentrations of Al (**A**), Fe (**B**), Cr (**C**), Pb (**D**), Ca (**E**), and Cd (**F**) uptaken by *T. domingensis* grown in iron mining tailings under different irrigation conditions. Different letters in columns indicate significant differences according to the Scott-Knott test at *P* < *0.05* (*n* = 10). Bars = standard error

Waterlogging also increased the essential element and pollutant uptake by *T. domingensis* (Fig. 2). The concentrations of Al were increased in the waterlogged treatment by 57.3% (Fig. 2A), 96.5% for Fe (Fig. 2B), 175.9% for Cr (Fig. 2C), 83.8% for Pb (Fig. 2D), 56.4% for Ca (Fig. 2E), and 105.7% for Cd (Fig. 2F) compared with field capacity.

**Fig. 3:**
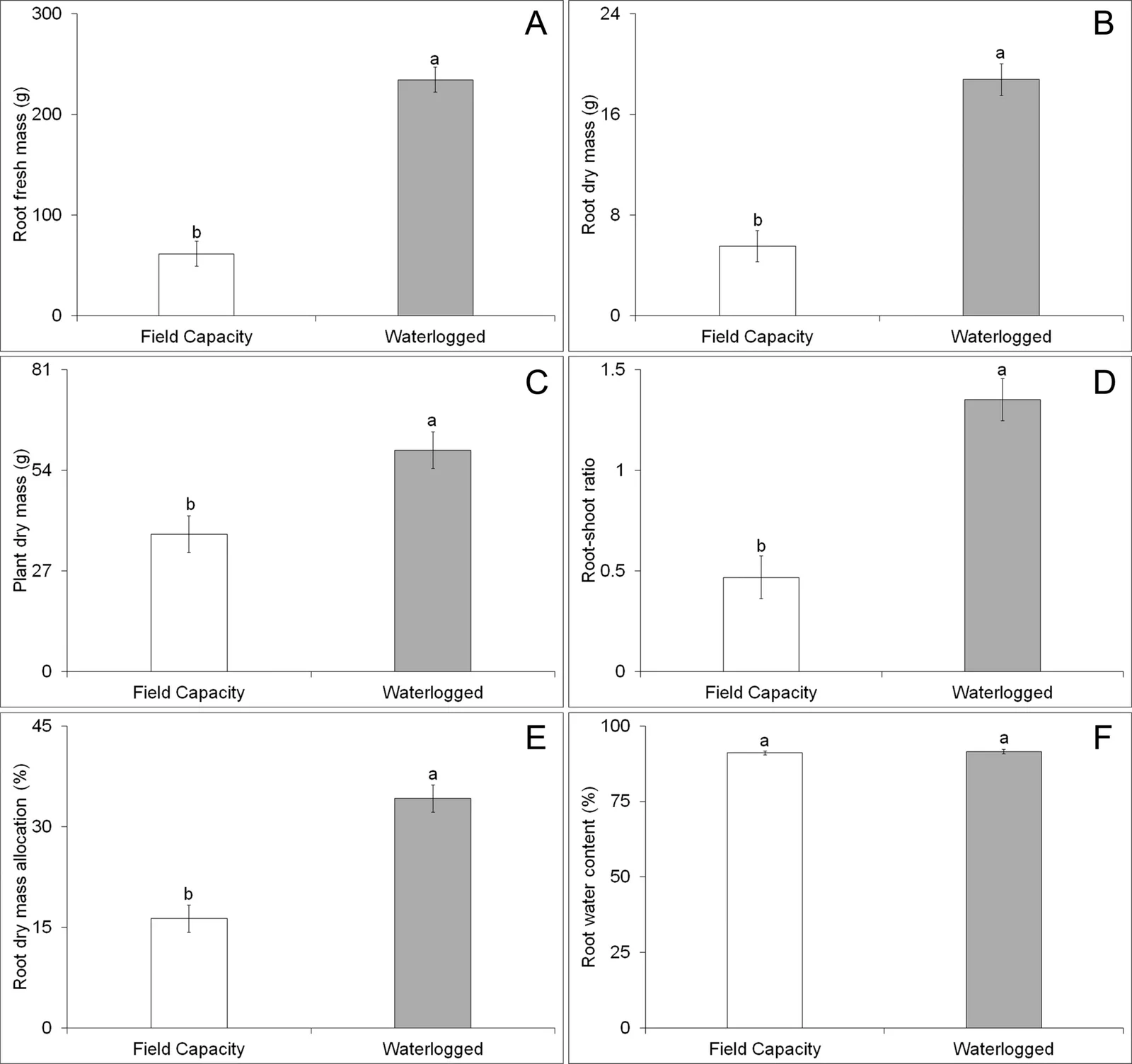
Growth and root traits from *T. domingensis* grown in iron mining tailings under different irrigation conditions. (**A**) root fresh mass, (**B**) root dry mass, (**C**) plant dry mass, (**D**) root:shoot ratio, (**E**) root dry mass allocation, (**F**) root water content. Different letters in columns indicate significant differences according to the Scott-Knott test at *P* < *0.05* (*n* = 18). Bars = standard error

**Fig. 4:**
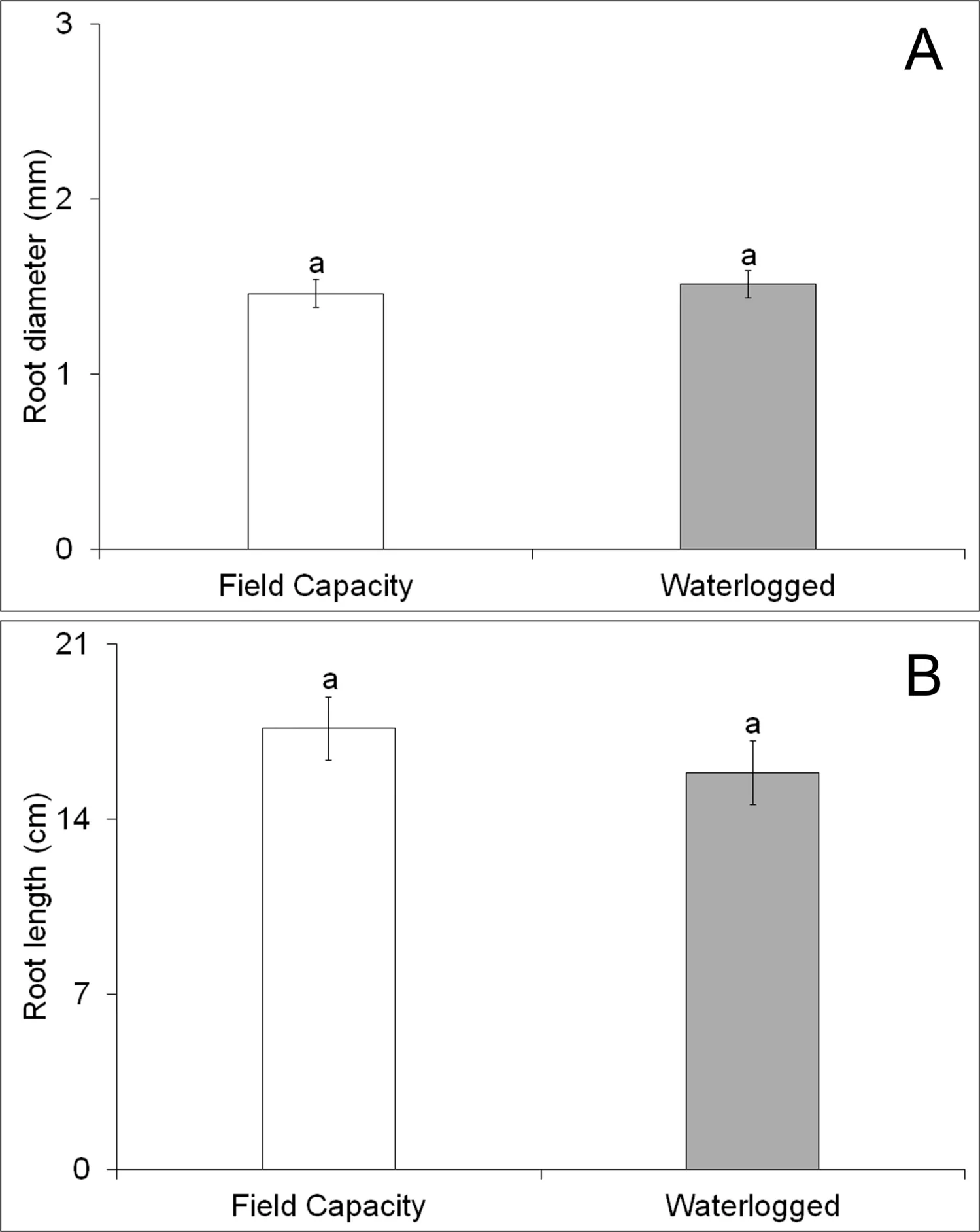
Diameter (**A**) and length (**B**) of *T. domingensis* roots grown in iron mining tailings under different irrigation conditions. Different letters in columns indicate significant differences according to the Scott-Knott test at *P* < *0.05* (*n* = 18). Bars = standard error

Waterlogging of iron mining tailings increased growth parameters from *T. domingensis* root fresh by 281.9% (Fig. 3A) and dry mass by 240.3% (Fig. 3B). Waterlogging also increased the total dry mass by 61.1% (Fig. 3C), the root:shoot ratio by 188.6% (Fig. 3D), and the root dry mass allocation by 110.1% (Fig. 3E). Nonetheless, waterlogging of iron mining tailings did not significantly affect some growth parameters such as the root water content (Fig. 3F, P = *0.6*), root diameter (Fig. 4A, P = *0.6*), or root length (Fig. 4B, P = *0.3*).

**Fig. 5:**
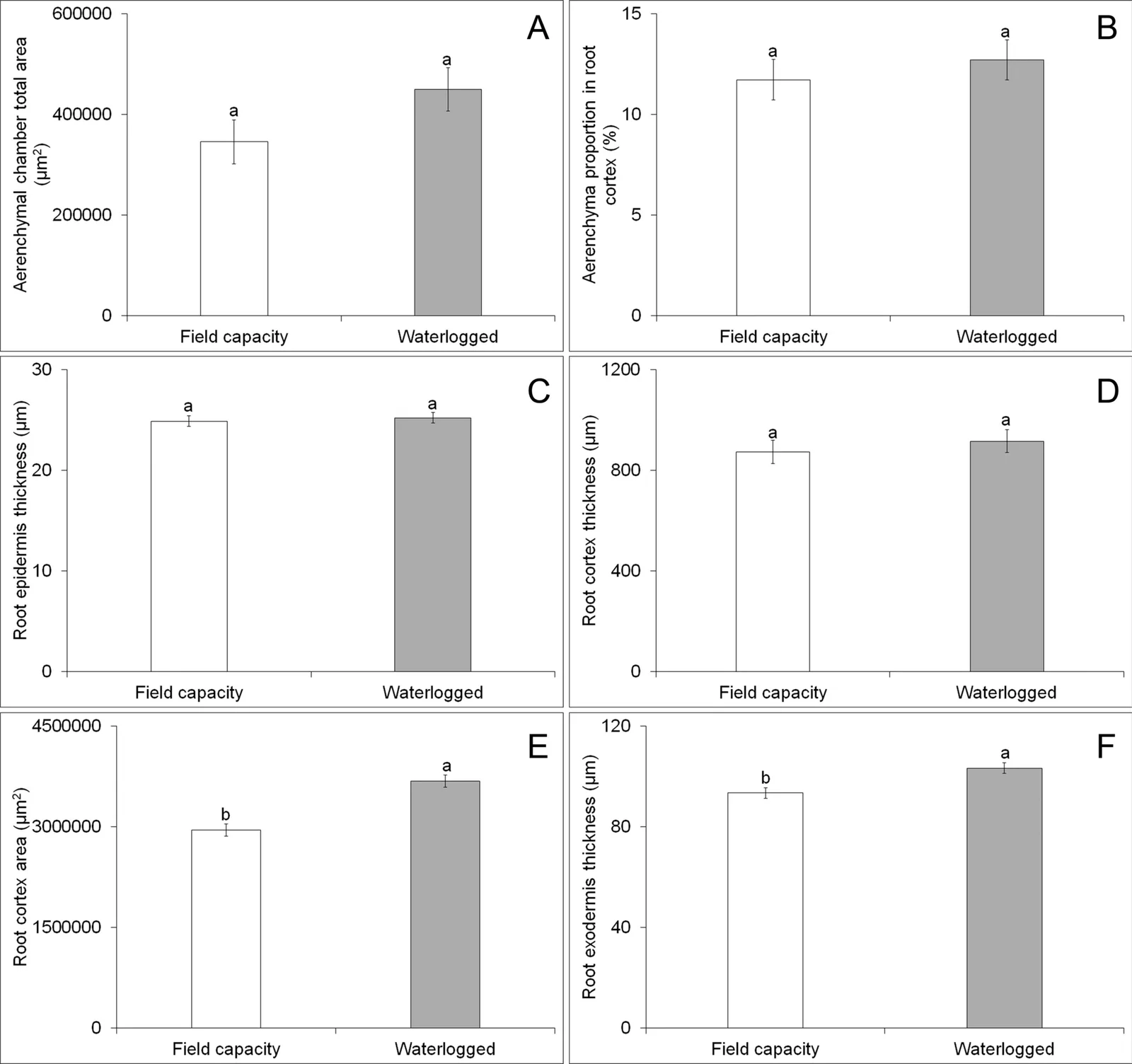
Root anatomical traits of *T. domingensis* grown in iron mining tailings under different irrigation conditions. **A** = aerenchyma chamber total area, **B** = aerenchyma proportion in the root cortex, **C** = root epidermis thickness, **D** = root cortex thickness, **E** = root cortex area, **F** = root exodermis thickness. Different letters in columns indicate significant differences according to the Scott-Knott test, *P* < *0.05* (*n* = 18). Bars = standard error

**Fig. 6:**
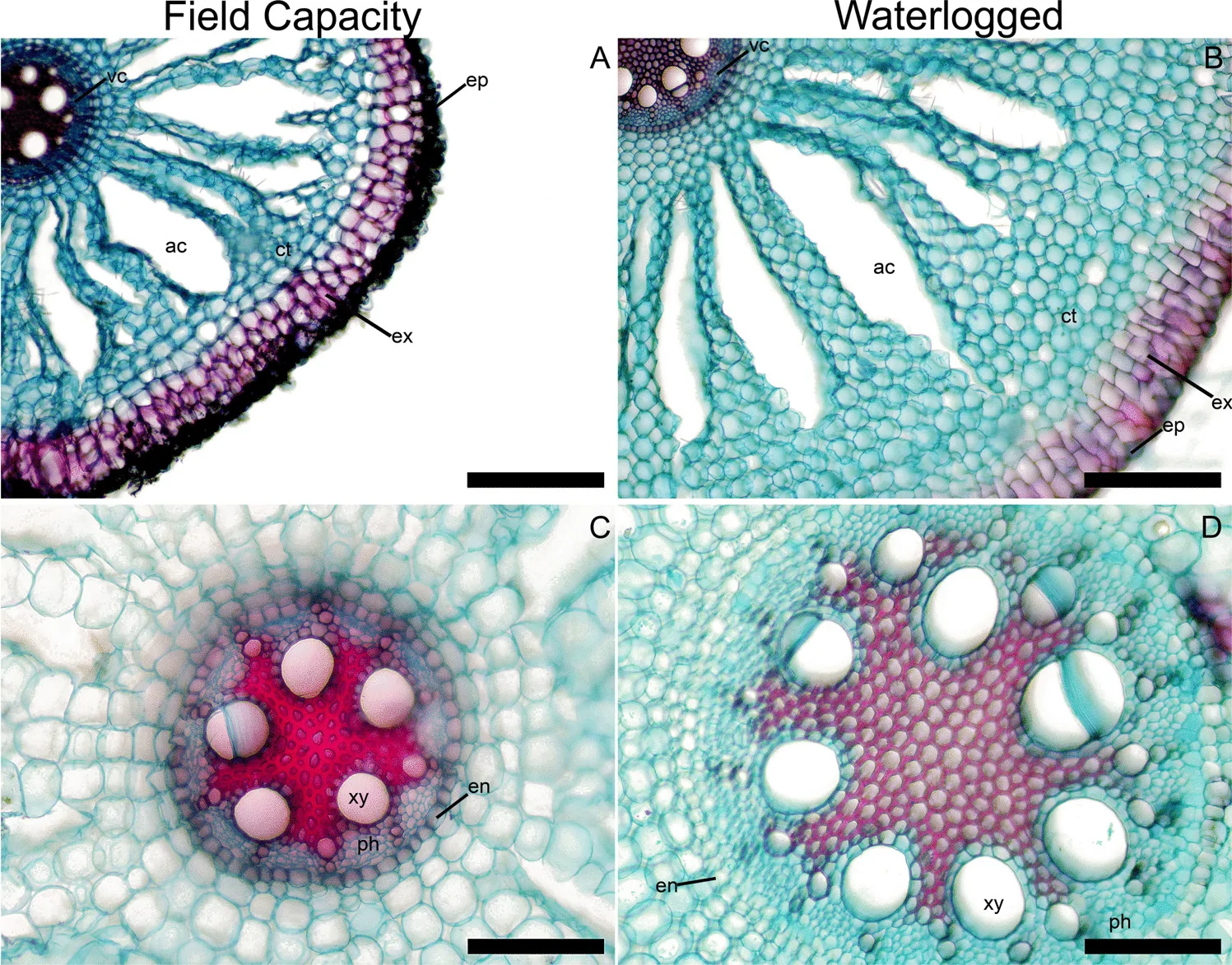
Transversal sections from roots of *T. domingensis* grown in iron mining tailings under different irrigation conditions. Field capacity (**A** and **C**), waterlogged (**B** and **D**). ep = epidermis; ct = cortex; ac = aerenchyma chamber; vc = vascular cylinder; ex = exodermis; en = endodermis; xy = xylem; ph = phloem. Bars: A and B = 250 μm; C and D = 125 μm

Waterlogging of iron mining tailings promoted no significant differences in the area of aerenchyma (Fig. 5A and Fig. 6, *P* = *0.1*), aerenchyma proportion (Fig. 5B and Fig. 6, *P* = *0.4*), epidermis thickness (Fig. 5C and Fig. 6, *P* = *0.6*), or the cortex thickness (Fig. 5D and Fig. 6, *P* = *0.5*) in *T. domingensis* roots. Waterlogging of iron mining tailings increased the cortex area by 24.6% (Fig. 5E and Fig. 6) and the exodermis thickness by 10.4% (Fig. 5F and Fig. 6) of *T. domingensis* roots.

**Fig. 7:**
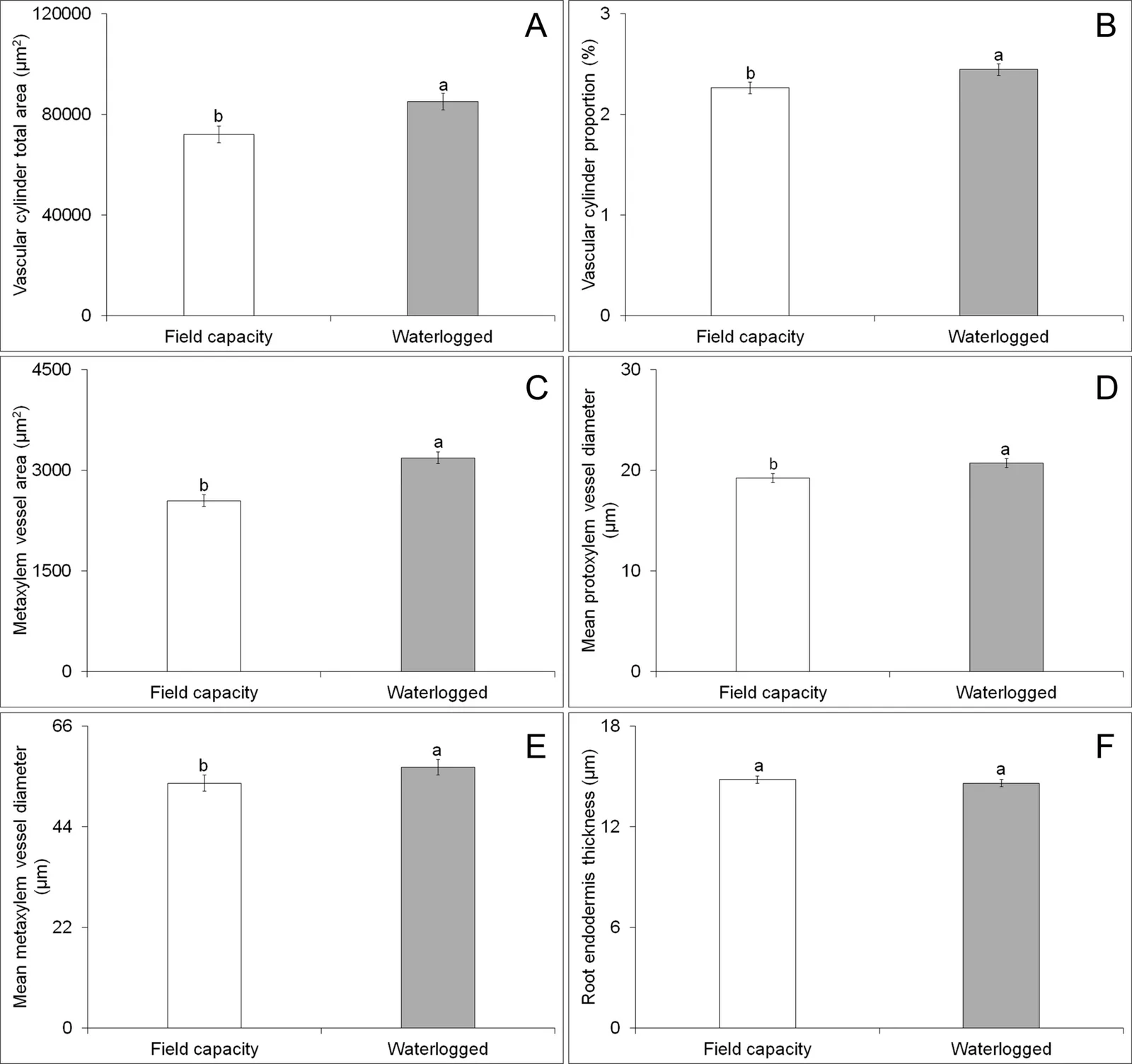
Anatomical parameters from the vascular cylinder of *T. domingensis* grown in iron mining tailings under different irrigation conditions. **A** = Vascular cylinder total area, **B** = vascular cylinder proportion, **C** = metaxylem vessel area, **D** = mean protoxylem vessel diameter, **E** = mean metaxylem vessel diameter, **F** = root endodermis thickness. Different letters in columns indicate significant differences according to the Scott-Knott test, *P* < *0.05* (*n* = 18). Bars = standard error

Waterlogging of iron mining tailings increased the vascular cylinder total area by 18.0% (Fig. 7A and Fig. 6), the vascular cylinder proportion by 8.1% (Fig. 7B and Fig. 6), the metaxylem vessel area by 24.9% (Fig. 7C and Fig. 6), the mean protoxylem vessel diameter by 7.7% (Fig. 7D and Fig. 6), and the mean metaxylem vessel diameter by 6.5% (Fig. 7E and Fig. 6) in roots of *T. domingensis*. Nonetheless, waterlogging of iron mining tailings promoted no significant differences for the root endodermis thickness (Fig. 7F and Fig. 6, *P* = *0.4*) of *T. domingensis*.

## Discussion

### Waterlogging of iron mining tailings increases the availability of essential element and pollutant to plants

Physicochemical parameters and pollutant uptake by *T. domingensis* grown suggest that waterlogging of iron mining tailings increases the availability of essential elements and pollutants. Water is the main solvent for ions present in soils, and a similar function can be attributed to iron mining tailings. Since ionic pollutants present in iron mining tailings are water-soluble (Dill et al. 2005), the increase in the volume of water in the waterlogged treatment favored the solubilization of ionic pollutants, increasing the electrical conductivity. In addition, potentially toxic elements can be more soluble under the lower redox potential of soils, promoted by waterlogging (Ponting et al. 2021). The higher amount of ions present in soils increases their electrical conductivity (Klein and Santamarina 2003). The increase in the total dissolved solids (TDS) observed in waterlogged conditions can also be related to increased solubilization of ions from iron mining tailings. The TDS values are related to all types of dissolved solids in water, including ions (Peng et al. 2020). Thus, the higher values for electrical conductivity may be related to the increased solubilization of ions present in iron mining tailings, which may include essential elements and pollutants with possible consequences for their toxicity.

The increase in the pH promoted by waterlogging of the iron mining tailings can be associated with alterations in the water redox potential, related to the solubilization of some elements and consumption of H^+^ from water. Waterlogging of soils favors reactions that require H^+^, increasing the pH values (Ponting et al. 2021). For instance, waterlogging of soils promotes the reduction of Fe and Mn, consuming H^+^ and increasing the soil's pH (Ponting et al. 2021). It is important to note that iron mining tailings' most abundant element is Fe (Table 1), providing a large amount of substrate for Fe reduction reactions, reducing the Fe^3+^ to Fe^2+^, which is more bioavailable being absorbed at higher amounts by plants. In addition, large Ca concentrations are found in iron mining tailings, being the third most abundant element (Table 1), and high Ca concentrations can also increase the pH of the soil. Ca is one of the most abundant elements in alkaline (high pH) soils (Jodral-Selgado et al. 2006), and Ca is used to consume H^+^ from acid soils, increasing their pH values (Mosley et al. 2024). The pH value of the tested iron mining tailings under field capacity (pH = 6, Fig. 1) is among the optimal for nutrient acquisition (Ponting et al. 2021; Hartermink and Barrow 2023; Mosley et al. 2024), and the higher values found in waterlogged treatments, being above 7.5 (Fig. 1), can reduce nutrient acquisition (Ponting et al. 2021; Hartermink and Barrow 2023; Gomes et al. 2025), affecting plant growth. Thus, higher Fe and Ca levels in the iron mining tailings can increase the pH of the pollutant when waterlogged, with potential impacts for nutrient acquisition.

The higher solubilization of ions in waterlogged iron mining tailings may have favored essential elements and pollutant availabilities, which were uptaken by plants at higher concentrations (Fig. 2). Several factors may explain the higher essential elements and pollutant availabilities in the waterlogged iron mining tailings. Waterlogging of soils reduces their redox potential, which makes Fe^2+^ ions more available to plants (Shaheen et al. 2014). The redox potential of waterlogged soils is reduced because O_2_ concentration is diminished in these conditions, being reduced to water (O_2_ + 4H^+^  + 4e^−^ = 2H_2_O), and since O_2_ shows the highest redox potential of the solution, and its depletion triggers NO_3_^−^ and MnO_2_ reduction, the redox potential drops significantly (Ohlsson 1979). In addition, long-term waterlogging of soils increases the availability of Cd and Cr (Resongles et al. 2015; Kelly et al. 2020). Reduced redox potential also increases Pb availability (Weber et al. 2009). Thus, ionic elements such as Al, Ca, Cd, Cr, Fe, and Pb may become more available at waterlogged iron mining tailings, being more absorbed by *T. domingensis;* however, further investigation is necessary on the identification and quantification of these elements in the water compared with their values in tailings, since it was not possible to evaluate these traits in this work.

### Tolerance of *T. domingensis* to waterlogged iron mining tailings

*Typha domingensis* grown under waterlogged iron mining tailings showed higher concentrations of pollutants (Fig. 2); however, the growth and development of these plants were not negatively affected by these conditions. These results indicate the tolerance of the species to these pollutants and also its potential for remediation of impacted areas by iron mining tailings.

The most abundant element present in iron mining tailings is Fe (Table 1), which is a micronutrient to plants, being required at small concentrations but may cause toxicity at high concentrations and low pH values, inhibiting lettuce germination (Gomes et al. 2025). *Typha domingensis* absorbed higher Fe concentrations in waterlogged iron mining tailings, but most of its growth parameters increased or remained unaffected by waterlogging (Fig. 3 and Fig. 4). This suggests a high tolerance to Fe concentrations by *T. domingensis,* which reached up to 3000 mg Kg^−1^ in the waterlogged treatment (Fig. 2). Excessive Fe concentrations reduce the growth of several species, such as *Brassica oleracea* L. (Peña-Olmos et al. 2014) and *Glycine max* L. Merr. (Gülser et al. 2019). Iron can be toxic for plants if accumulated in concentrations of 500 mg Kg^−1^ in plant tissues, causing growth limitations (Lapaz et al. 2022). In addition, Fe toxicity increases at pH values of 5 or lower (Lapaz et al. 2022; Gomes et al. 2025). *T. domingensis* accumulated Fe at higher values in both irrigation treatments in iron mining tailings (Fig. 2), suggesting a high Fe-tolerance and accumulation capacity of this species; however, the pH values of the iron mining tailings in both irrigation treatments were above 6, and further investigation is necessary to understand the effect of lower pH values on Fe toxicity in waterlogged iron mining tailings.

The element with the second-highest concentration in iron mining tailings was Ca (Table 1), and also the second most absorbed by plants (Fig. 2). Ca is a macronutrient, being essential to several processes in plants, such as cell signaling and as a component of cell walls (White and Broadley 2003). The Ca demand for *T. domingensis* is still unclear; however, at the levels accumulated by these plants growing in iron mining tailings, it seems not to harm the growth of the species remaining at the levels demanded as a nutrient.

The third most abundant element in iron mining tailings is Al (Table 1), and it was also the third most absorbed by *T. domingensis* (Fig. 2). Al is a non-essential element for plants, and it is among the most toxic for roots (Kochian et al. 2015). The higher uptake of Al in waterlogged iron mining tailings did not harm *T. domingensis* growth, since plants showed increased growth parameters under these conditions, suggesting tolerance to Al toxicity. Negative effects of Al on plant root growth are extensively reported in the literature, as demonstrated for *Oryza sativa* L. (Kikui et al. 2005), *Secale cereale* L. (Ma et al. 2002), and *Zea mays* L. (Batista et al. 2013). Al toxicity increases significantly at pH values of 5 or lower (Kochian et al. 2015; Gomes et al. 2025). *T. domingensis* was tolerant to high concentrations of Al found in iron mining tailings and absorbed significant concentrations (Table 1 and Fig. 2); however, since the pH of the iron mining tailings remained above 6 in both irrigation treatments (Fig. 1), further investigation is necessary on the effect of lower pH values in waterlogged iron mining tailings for Al toxicity.

Other pollutants found in iron mining tailings include Cd, Cr, and Pb, which showed lower concentrations (Table 1) but were absorbed at higher levels in waterlogged conditions (Fig. 2). Despite the concentrations of Cd, Cr, and Pb being at lower levels, these are toxic metals with high toxicity levels (Krämer 2010). Despite being absorbed in both irrigation treatments and at higher levels in waterlogged iron mining tailings, these pollutants did not harm *T. domingensis* growth, suggesting tolerance for these elements and potential for remediation of iron mining tailings. Cd toxicity reduces plant growth and dry mass production as shown for *Zea mays* (Perveen et al. 2015) and *Hordeum vulgare* L. (Nouri et al. 2019). Cr uptake also diminishes the dry mass production in several plants, including *Camellia sinensis* L. (Tang et al. 2012), *Raphanus sativus* L. (Tiwari et al. 2013), *Zea mays,* and *Brassica nigra* (L.) Koch (Kant et al. 2018), and harms the root growth of *Citrus aurantium* L. (Shiyab 2019). Pb uptake also reduces the growth and dry mass of plant species, such as *Brassica oleracea* (Sinha et al. 2006), *Sorghum bicolor* L.*,* and *Glycine max* (Cândido et al. 2020). Most toxic metals harm plant and root growth because of the production of reactive oxygen species, which damage the photosynthetic system (Khorobrykh et al. 2020). *Typha domingensis* was already reported as tolerant to Cd (de Oliveira et al. 2018, 2022), and Cr (Mufarrege et al. 2015), and *Typha angustifolia* L. species is tolerant to lead (Bah et al. 2010).

Thus, results suggest that *T. domingensis* is tolerant to multiple elements included in iron mining tailings (Al, Ca, Fe, Cd, Cr, and Pb), and despite these elements becoming more available in waterlogged conditions and being absorbed by plants at higher concentrations in these conditions, the absence of damage to its growth indicates the potential of this species for the remediation of iron mining tailings.

### Role of root anatomy in *T. domingensis* tolerance to iron mining tailings

Most of the root anatomy of *T. domingensis* was not significantly changed by irrigation conditions (Fig. 5 and Fig. 6), and no evidence of necrosis or deformations was found when compared with previous works on the anatomical traits of the plant, for instance, Duarte et al. (2021) and de Oliveira et al. (2022). Preserving the root anatomy when grown in iron mining tailings is an important tolerance trait for *T. domingensis.*

Some of the root anatomical traits modified by waterlogged iron mining tailings can be related to *T. domingensis* tolerance to this pollutant. The thicker exodermis developed under waterlogged iron mining tailings by *T. domingensis* can act as an apoplastic barrier, avoiding pollutant intake at concentrations above which the plant can tolerate. The exodermis is a tissue located internally to the epidermis (Fig. 6), which contains cells with suberized or lignified cell walls (Liu and Kreszies 2023; Manzano et al. 2025). Tolerant species such as *T. domingensis* can increase root tissues to reduce the uptake of the pollutant (Oliveira et al. 2022). Thus, results suggest that a thicker exodermis in *T. domingensis* roots is a trait that may be a potential mechanism to avoid excessive pollutant uptake since waterlogging increases their availability; however, this mechanism should be further investigated regarding the amount of pollutants in the solution and the actual capacity of the tissue to constrain their uptake.

The larger cortex area in *T. domingensis* roots grown under waterlogged mining tailings suggests that it can be an important tolerance trait, because increasing the cortex may improve the capacity to retain pollutants in the plant's roots. The root cortex comprises an interface between the environment and the vascular cylinder (Jones et al. 2025), after water and pollutants have passed through the epidermis. *Typha domingensis* tolerance to Cd also includes protection of the photosynthetic tissues by storing the pollutant mostly in their roots (de Oliveira et al. 2018). Thus, results suggest that the increase in the root cortex of *T. domingensis* may favor the accumulation of pollutants present in waterlogged iron mining tailings in their roots, but this aspect must be further investigated.

## Conclusion

This work hypothesizes that waterlogging increased the availability of essential elements and pollutants present in iron mining tailings, but *Typha domingensis* Pers. can survive under these conditions, showing potential for phytoremediation. Waterlogging of iron mining tailings increases the uptake of Al, Ca, Cd, Cr, Fe, and Pb by *T. domingensis* because of a larger water volume and these elements being solubilized and becoming more available. The root exodermis and cortex were increased in waterlogged tailings, thickening the apoplastic barriers and the tissues in roots to store pollutants. The organ-specific allocation of pollutants and the tolerance mechanisms to alkaline conditions (pH > 7) of *T. domingensis* grown in waterlogged iron mining tailings are unknown and must be investigated in the future. Regardless of absorbing more essential elements and pollutants in waterlogged iron mining tailings, *T. domingensis* growth was stimulated by waterlogging, supporting the potential of the species for phytoremediation of impacted areas.

## Data Availability

The datasets used for analyses during the current study are available from the corresponding author upon reasonable request.
